# Conditional Generative Adversarial Networks for Individualized Treatment Effect Estimation and Treatment Selection

**DOI:** 10.3389/fgene.2020.585804

**Published:** 2020-12-11

**Authors:** Qiyang Ge, Xuelin Huang, Shenying Fang, Shicheng Guo, Yuanyuan Liu, Wei Lin, Momiao Xiong

**Affiliations:** ^1^Department of Biostatistics and Data Science, School of Public Health, The University of Texas Health Science Center at Houston, Houston, TX, United States; ^2^School of Mathematical Sciences, Fudan University, Shanghai, China; ^3^Department of Biostatistics, The University of Texas MD Anderson Cancer Center, Houston, TX, United States; ^4^Department of Surgical Oncology, The University of Texas MD Anderson Cancer Center, Houston, TX, United States; ^5^Department of Medical Genetics, University of Wisconsin-Madison, Madison, WI, United States

**Keywords:** causal inference, generative adversarial networks, counterfactuals, treatment estimation, precision medicine

## Abstract

Treatment response is heterogeneous. However, the classical methods treat the treatment response as homogeneous and estimate the average treatment effects. The traditional methods are difficult to apply to precision oncology. Artificial intelligence (AI) is a powerful tool for precision oncology. It can accurately estimate the individualized treatment effects and learn optimal treatment choices. Therefore, the AI approach can substantially improve progress and treatment outcomes of patients. One AI approach, conditional generative adversarial nets for inference of individualized treatment effects (GANITE) has been developed. However, GANITE can only deal with binary treatment and does not provide a tool for optimal treatment selection. To overcome these limitations, we modify conditional generative adversarial networks (MCGANs) to allow estimation of individualized effects of any types of treatments including binary, categorical and continuous treatments. We propose to use sparse techniques for selection of biomarkers that predict the best treatment for each patient. Simulations show that MCGANs outperform seven other state-of-the-art methods: linear regression (LR), Bayesian linear ridge regression (BLR), k-Nearest Neighbor (KNN), random forest classification [RF (C)], random forest regression [RF (R)], logistic regression (LogR), and support vector machine (SVM). To illustrate their applications, the proposed MCGANs were applied to 256 patients with newly diagnosed acute myeloid leukemia (AML) who were treated with high dose ara-C (HDAC), Idarubicin (IDA) and both of these two treatments (HDAC+IDA) at M. D. Anderson Cancer Center. Our results showed that MCGAN can more accurately and robustly estimate the individualized treatment effects than other state-of-the art methods. Several biomarkers such as GSK3, BILIRUBIN, SMAC are identified and a total of 30 biomarkers can explain 36.8% of treatment effect variation.

## Introduction

Traditional clinical management estimates the average treatment effects from observational data, assuming that the complex disease is homogeneous (Rosenbaum and Rubin, 1983; Hansen, 2004; Diamond and Sekhon, 2013; Kennedy et al., 2017; Liu et al., 2018; Luo and Zhu, 2020). Alternatives to traditional clinical management, “precision medicine” or “precision oncology” attempts to match the most accurate and effective treatments with the individual patient (Shin et al., 2017; Ali and Aittokallio, 2019), rather than using monotherapy that treats all patients. In the real world, treatment response is heterogeneous. Therapy should be tailored with the best response possible and highest safety margin to ensure that the right therapy is offered to “the right patient at the right time” (Subbiah and Kurzrock, 2018). Precision oncology can substantially improve progress and treatment outcomes of patients. It plays a central role in revolutionizing cancer research. Consequently, alternative to calculating the average effect of an intervention over a population, many recent methods attempt to estimate individualized treatment effects (ITEs) or conditional average treatment effects from observational data (Makar et al., 2019). To accurately estimate the individualized treatment effects and learn optimal treatment choices are key issues for precision oncology. More accurate estimation of individualized treatment effects, which provides information to guide the individual selection of the target therapies, is essential for the success of precision medicine (Kornblau et al., 2009).

Methods for estimation of individualized treatment effects (ITEs) using observational data largely differ from standard statistical estimation methods. Estimating of ITEs and learning optimal treatment strategies raise a great challenge for the following reasons. First, a common framework for treatment effect estimation is the potential outcomes assumptions (Ray and Szabo, 2019) where every individual has two “potential outcomes” covering the hypothesized individual's outcomes with and without treatment. Estimation of ITEs requires estimation of both factual and counterfactual outcomes for each individual. However, only the factual outcome is actually observed. We never observe the counterfactual outcomes (Rosenbaum and Rubin, 1983; Chen and Paschalidis, 2018; Yoon et al., 2018a).

If the effect of each treatment in the subpopulation which is separately estimated is taken as an individual effect, this can create large biases. The estimated effect of each treatment in the subpopulation is still the average effect of the treatment in that subpopulation and is not an individualized treatment effect in the subpopulation.

Second, clinical data often have many missing values. Simultaneously imputing both counterfactual values and missing values is not easy. Third, the function forms of the treatment effects which are often non-linear functions are unknown (Ray and Szabo, 2019). Statistical methods and computational algorithms that can efficiently deal with unknown forms of non-linear functions are still lacking (Lengerich et al., 2019).

Classical works such as random forest and hierarchical models are adapted to estimate heterogeneous treatment effects (Wager and Athey, 2015). Recently, machine learning and neural network methods are used to move away from average treatment effect estimation to personalized estimation (Johansson et al., 2016; Shalit et al., 2016; Alaa and van der Schaar, 2017). AI and causal inferences are becoming a driving force for innovation in precision oncology (Seyhan and Carini, 2019). A key issue for ITE estimation is to learn unobserved (missing) counterfactuals. The idea of using generative adversarial networks (GANs) for handling missing data is a very promising approach to imputing counterfactual (Goodfellow et al., 2014; Ding and Li, 2017; Yoon et al., 2018a). Using conditional GAN (CGAN) to estimate the individualized treatment effects (GANITE) has been developed (Yoon et al., 2018a,b). The CGANs consist of a generator and a discriminator. The generator (G) observes the factual part of real data and imputes the counterfactuals (missing part) conditioned on observed factual data, and outputs the complete dataset. The discriminator (D) inputs the real dataset and tries to determine which part was actually observed and which part was imputed counterfactuals. The discriminator enforces the generator to learn the desired distribution (hidden data distribution) (Yoon et al., 2018b).

However, the original GANITE was designed for estimation of the effects of binary treatment and cannot be applied to continuous and categorical treatments. The treatment variable in the original GANITE is a binary variable which only represents the presence and absence of treatment. Therefore, the treatment variable in the original GANITE is unable to quantify the dosage of the treatment, and hence the original GANITE cannot be applied to continuous treatment. To overcome this limitation, we introduce a treatment assignment indicator variable and treatment quantity variable. The treatment quantity variable can represent binary treatment, categorical treatment, and continuous treatment. We change mathematical formulations of the generator and discriminator and extend GANITE from binary treatment to all types of treatments including binary, categorical, and continuous treatments. The modified GANITE is abbreviated as MGANITE.

GANITE or in general, CGAN has not systematically investigated the estimation of ITE for chemotherapy and other types of treatments in cancer and compared the results from causal inference using observed data with the results of randomized clinical trials. One of our goals in this manuscript is to examine whether MGANITE still works well in cancer research.

In MGANITE, biomarkers that serve as conditioned variables, will be used to estimate the ITEs of both single and multiple treatments (Mirza and Osindero, 2014; Yoon et al., 2018a). Sparse techniques will be employed to select biomarkers for prediction of treatment effects and to learn optimal treatment choices of patients (Emmert-Streib and Dehmer, 2019).

In summary, The novelty of modified GANITE (MGANITE) is summarized below.

1. The previous conditional generative adversarial network (CGAN)-based causal inference methods (GANITE) only can estimate the individualized effects of binary treatment and cannot estimate the individualized effects of continuous treatments. The proposed MGANITE is the first time to use modified CGANs for estimation of individualized effects of continuous treatments.
2. We develop new network structures for the generator and discriminator in the CGANs.
3. We combined sparse techniques for selection of biomarkers with MGANITE to predict the best treatment for each patient.

To evaluate its performance for estimating ITEs, simulations are conducted to estimate ITEs using simulated data and MGANITE, and to compare its estimation accuracy with five other state-of-the-art methods (LR, KNN, BLR, random forest, and SVM). To further evaluate its performance, MGANITE is applied to 256 newly diagnosed acute myeloid leukemia (AML) patients, treated with high dose ara-C (HDAC), Idarubicin (IDA), and HDAC+IDA at M. D. Anderson Cancer Center to estimate ITEs and identify the optimal treatment strategy for each patient. Preliminary results from simulations and real data analysis show that MGANITE outperforms five other state-of-the-art methods. A program for implementing the proposed MGANITE for ITE estimation and optimal treatment selection can be downloaded from our website https://sph.uth.edu/research/centers/hgc/software/xiong/.

## Materials and Methods

### Potential Outcome Framework for Estimation of Treatment Effects

We assume the Rubin causal model for estimation of treatment effects (Rubin, 1974) and modifies the approach to the individualized treatment effect estimation in Yoon et al., 2018a). The original GANITE only can estimate ITE of binary treatments, but it cannot be applied to categorical and continuous treatments. We develop MGANITE which can estimate ITE of all types of treatments including binary, categorical, and continuous treatments by introducing a treatment assignment indicator variable and changing the formulation of the generator and discriminator. Consider *K* treatments. Let *T*_*k*_ be the *k*^*th*^ treatment variable that can be binary, categorical or continuous, and $T={\left[T_{1},\ldots ,T_{K}\right]}^{T}$ be the treatment vector. We assume that there is precisely one non-zero component of the treatment vector *T*, which is denoted by *T*_η_, where η is the index of this component. Each sample has one and only one assigned treatment *T*_η_. To extend the binary treatment to include categorical and continuous treatments, we define the treatment assignment indicator vector $M={\left[M_{1},\ldots ,M_{k},\ldots ,M_{K}\right]}^{T}$ as

$$\begin{array}{l} M_{k}=\left\{ \begin{array}{c} 1\;k=\eta \\ 0\text{ otherwise} \end{array}\right. \end{array}$$

where $\overset{K}{\underset{k=1}{\sum }}M_{k}=\;1$.

For example, if

$$\begin{array}{l} T=\left[\begin{array}{c} 0\\ T_{2}\\ 0 \end{array}\right] \end{array}$$

then η = 2 and

$$\begin{array}{l} M=\left[\begin{array}{c} 0\\ 1\\ 0 \end{array}\right] \end{array}$$

If we consider treated and untreated cases, then *K* = 2. Let *T*_1_ denote the treatment and *T*_2_ denote no treatment where *T*_2_ = 1. For the sample with the treatment, we have

$$\begin{array}{l} T=\left[\begin{array}{c} T_{1}\\ 0 \end{array}\right]\text{ and }M=\left[\begin{array}{c} 1\\ 0 \end{array}\right] \end{array}$$

For the sample with no treatment, we have

$$\begin{array}{l} T=\left[\begin{array}{c} 0\\ T_{2} \end{array}\right]\text{ and }M=\left[\begin{array}{c} 0\\ 1 \end{array}\right]. \end{array}$$

Define the vector of potential outcome $Y\left(T\right)={\left[Y\left(T_{1}\right),\ldots ,\;Y\left(T_{K}\right)\right]}^{T}$, where *Y*(*T*_*k*_) is the potential outcome of the sample under the treatment *T*_*k*_. When *K* = 2, the potential outcome *Y*(*T*_1_) corresponds to the widely used notation for one treatment *Y*^1^, the potential outcome of the treated sample, while the potential outcome *Y*(*T*_2_) corresponds to *Y*^0^, the potential outcome of the untreated sample. Only one of the potential outcomes can be observed. The observed outcome that corresponds to the potential outcome of the individual receiving the treatment *T*_η_ is denoted by *Y*(*T*_η_). The observed outcome is called the factual outcome and the unobserved potential outcomes are called counterfactual outcomes, or simply counterfactuals. For the convenience of notation, the factual outcome is also denoted by *Y*_*f*_ and the counterfactuals are denoted by *Y*_*cf*_.

The observed outcome *Y*_*f*_ can be expressed as

$$\begin{array}{l} Y_{f}=Y_{\eta }=\overset{K}{\underset{k=\;1}{\sum }}M_{k}Y\left(T_{k}\right) \end{array}$$

When *K* = 2, we have *M*_2_ = 1−*M*_1_. The above equation becomes

$$\begin{array}{l} Y_{f}=M_{1}Y\left(T_{1}\right)+\left(1-M_{1}\right)Y\left(T_{2}\right)=M_{1}Y^{1}+\left(1-M_{1}\right)Y^{0} \end{array}$$

which coincides with the standard expression of the observed outcome for one treatment.

Let $X={\left[X_{1},\ldots ,\;X_{q}\right]}^{T}$ be the *q*-dimensional feature vector. Assume that *n* individuals are sampled. Let $T^{\left(i\right)}={\left[T_{1}^{\left(i\right)},\;\ldots ,\;T_{K}^{\left(i\right)}\right]}^{T},\;Y^{\left(i\right)}={\left[Y^{\left(i\right)}(T_{1}^{\left(i\right)}),\;\ldots ,\;Y^{\left(i\right)}(T_{K}^{\left(i\right)})\right]}^{T}$ and $X^{\left(i\right)}={\left[X_{1}^{\left(i\right)},\;\ldots ,\;X_{q}^{\left(i\right)}\right]}^{T},\;i=1,\ldots ,\;n$ be the treatment vector, the vector of potential outcomes, and feature vector of the *i*^*th*^ individual, respectively.

The most widely used measure of the treatment effect for the multiple treatment is the pair-wise treatment effect. The individual effect $\xi_{jk}^{\left(i\right)}$ between the pairwise treatments: *T*_*j*_ and *T*_*k*_ is defined as $\xi_{jk}^{\left(i\right)}=Y^{\left(i\right)}\left(T_{j}^{\left(i\right)}\right)-Y^{\left(i\right)}\left(T_{k}^{i}\right)$, the average pairwise treatment effect $\tau_{jk}=E\left[\xi_{jk}^{\left(i\right)}\right]$. The average pairwise treatment effect τ_*jk*|_*T*__*j*__ on the patients treated with *T*_*j*_ is defined as $\tau_{jk|T_{j}}=E\left[\xi_{jk}^{\left(i\right)}|T_{j}\;\right]$.

The focus of this paper is on the conditional distribution of treatment effect, given the feature vector *X*. Let *F*_*Y*|*X*_(*T*_*k*_) be the conditional distribution of the potential outcome *Y*(*T*_*k*_) under the treatment *T*_*k*_, given the feature vector *X*, and *F*_*Y*|*X*_(*T*) be the conditional joint distribution of the potential outcome vector *Y*(*T*) under the *K* treatment *T*, given the feature vector *X*. Assume that *n* individuals are sampled. For the *i*^*th*^ individual, *T*_η_ treatment (*M*_η_ = 1) is assigned. Let *X*^(*i*)^ and $Y_{\eta }^{\left(i\right)}\left(T_{\eta }^{\left(i\right)}\right)=Y_{f}^{\left(i\right)}$ be the observed feature vector and the observed potential outcome of the *i*^*th*^ individual. Therefore, the observed dataset is given by $D=\left(X^{\left(i\right)},\;T^{\left(i\right)},\;Y_{\eta }^{\left(i\right)},\;i=1,\ldots ,n\right)$. The factual and counterfactual outcomes of the *i*^*th*^ individual are denoted by $y_{f}^{\left(i\right)}$ and $y_{cf}^{\left(i\right)}$, respectively.

To estimate the treatment effects, we often make the following three assumptions (Rubin, 1974; Yoon et al., 2018a):

Assumption 1 (Ignorability Assumption). Conditional on *X*, the potential outcomes, *Y*(*T*) and the treatment *T* are independent,

(1) $$\begin{array}{l} Y\left(T\right)=\left(Y\left(T_{1}\right),\ldots ,\;Y\left(T_{K}\right)\right)T|X \end{array}$$

This assumption requires no unmeasured confounding variables.

Assumption 2 (Common Support). For the feature vector *X* and all treatment,

(2) $$\begin{array}{l} 0<P\left(T_{k}=t_{k}|X\right)<1 \end{array}$$

Assumption 3 (Stable Unit Treatment Value Assumption). No interference (units do not interfere with each other).

### Conditional Generative Adversarial Networks as a General Framework for Estimation of Individualized Treatment Effects

The key issue for the estimation of individualized treatment effects is unbiased counterfactual estimation. Counterfactuals will never be observed and cannot be tested by data. The true counterfactuals are unknown. Recently developed generative adversarial networks (GANs) started a revolution in deep learning (Luo and Zhu, 2020). GANs are a perfect tool for missing data imputation. An incredible potential of GANs is to accurately generate the hidden (missing) data distribution given some of the features in the data. Therefore, we can use GANs to generate counterfactual outcomes.

GANs consist of two parts: the “generative” part that is called the generator and “adversarial” part that is called the discriminator. Both the generator and discriminator are implemented by neural networks. Typically, a *K*-dimensional noise vector is input into the generator network that converts the noise vector to a new fake data instance. Then the generated new data instance is input into the discriminator network to evaluate them for authenticity. The generator constantly learns to generate better fake data instances while the discriminator constantly obtains both real data and fake data and improves accuracy of evaluation for authenticity.

#### Architecture of Conditional Generative Adversarial Networks (CGANs) for Generating Potential Outcomes

**Figure 1:**
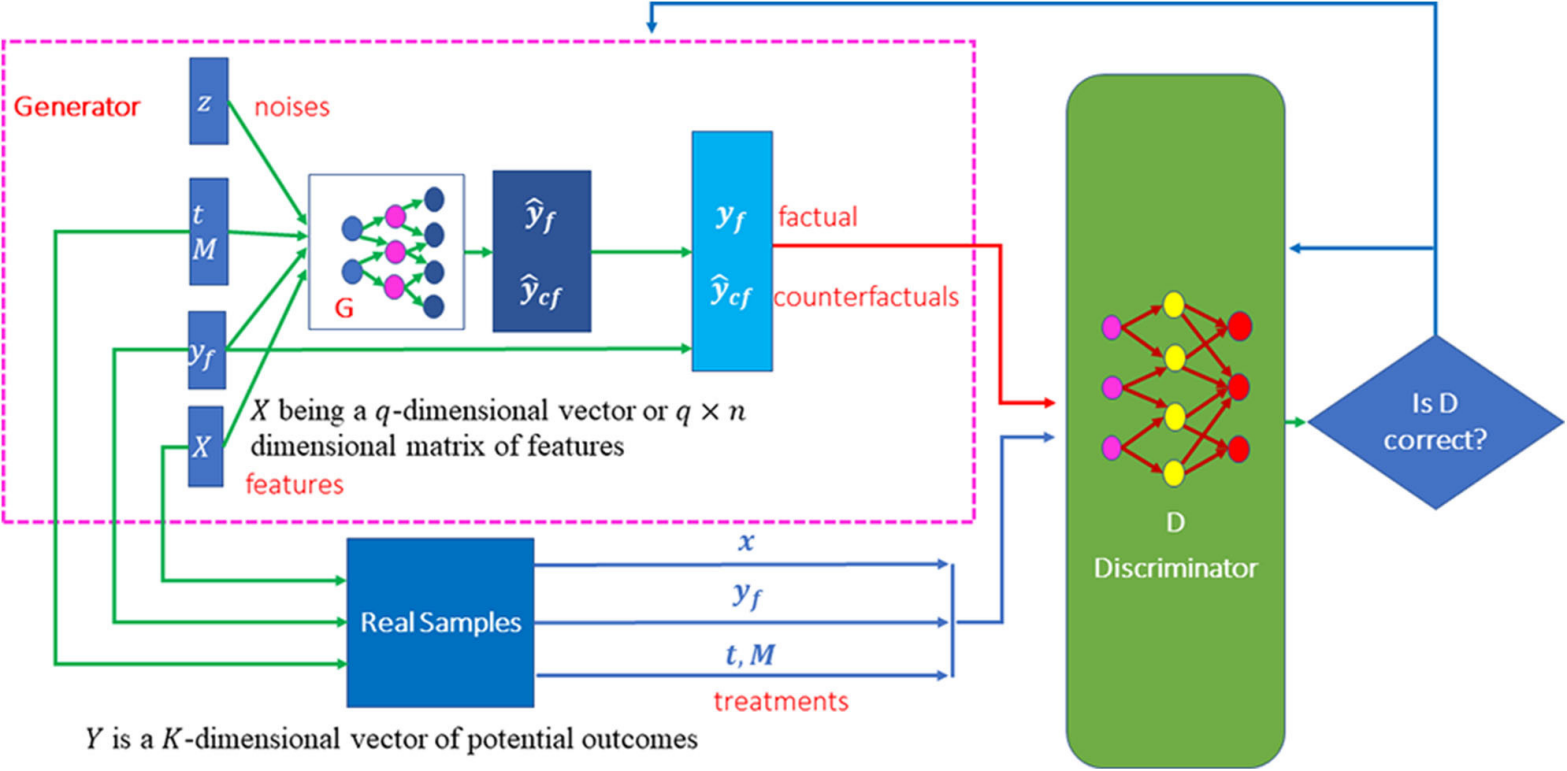
Scheme of MGANITE for the estimation of potential outcomes.

Features provide essential information for estimation of counterfactual outcomes. Therefore, we use conditional generative adversarial networks (CGANs) (Mirza and Osindero, 2014) as a general framework for individualized treatment effect (ITE) estimation. The CGANs for ITE estimation consist of two blocks. The first imputation block is to impute the counterfactual outcomes. The second ITE block is to estimate distribution of the treatment effects using the complete dataset that is generated in the imputation block. The architecture of CGANs is shown in Figure 1.

Both the generator and discriminator are implemented by feedforward neural networks. The architectures of the neural networks are described as follows. The generator consists of seven layers of feedforward neural network. The first layer is the covariate input layer that input a vector *X* of covariates. The second and third layers are hidden layers, each layer with 64 nodes. The fourth layer concatenates the output of the third layer, the response vector Y, treatment vector T and treatment assignment indicator vector M and noise vector Z. The fifth and sixth layers are hidden layers, each layer with 64 nodes. Finally, the seventh layer is the output layer. All activation functions of the neurons were sigmoid function. The architecture of the discriminator is similar to the architecture of the generator except for adding one more output layer with sigmoid non-linear activation function.

#### Imputation Block

To extend GANTITE from binary treatments to all types of treatments, we introduce the treatment assignment vector and change some mathematical formulation of the generator. A counterfactual generator in the imputation block is a non-linear function of the feature vector, treatment vector *T*, treatment assignment indicator vector *M*, observed factual outcome *y*_*f*_ and *K* dimensional random vector *z*_*G*_ with uniform distribution $z_{G}\sim U\left({\left(-1,1\right)}^{K}\right)$ where *Y*_*f*_ = *Y*_η_. The generator is denoted by

(3) $$\begin{array}{l} \tilde{Y}=G\left(X,Y_{f},{T⊙M,\;\left(1-M\right)⊙z}_{G},\theta_{G}\right) \end{array}$$

where output Ỹ represents a sample of *G*. It can take binary values, categorical values or continuous values. **1** is a vector of 1, ⊙ denotes element-wise multiplication, and θ_*G*_ is the parameters in the generator. We use $\bar{}Y$ to denote the complete dataset that is obtained by replacing Ỹ_η_with *Y*_*f*_.

The distribution of Ỹ depends on the determinant of the Jacobian matrix of the transformation function *G*(*X, Y*_*f*_, *T, M, z*_*G*_, θ_*G*_). Changing the transformation function can change the distribution of the generated counterfactual outcomes. Let *P*_*Y*|*x, t*,_*m, y*__*f*__(*y*) be the conditional distribution of the potential outcomes, given *X* = *x, T* = *t, M* = *m, Y*_*f*_ = *y*_*f*_. The goal of the generator is to learn the neural network *G* such that *G*(*x, y*_*f*_, *t, m, z*_*G*_, θ_*G*_)~*P*_*Y*|*x, t*,_*m, y*__*f*__(*y*).

Unlike the discriminator in the standard CGANs where the discriminator evaluates the input data for their authenticity (real or fake data), the counterfactual discriminator *D*_*G*_ that maps pairs (*x*,*y*) to vectors in [0, 1]^*k*^ attempts to distinguish the factual component from the counterfactual components. The output of the counterfactual discriminator *D*_*G*_ is a vector of probabilities that the component represents the factual outcome. Let *D*_*G*_(*x*, ỹ, *t, m*,_θ_*d*_)*i*_ represent the probability that the *i*^*th*^component of ỹ is the factual outcome, i.e., *i* = η, where θ_*d*_ denotes the parameters in the discriminator. The goal of the counterfactual discriminator is to maximize the probability *D*_*G*_(*x*, ỹ, *t, m*,_θ_*d*_)*i*_ for correctly identifying the factual component η via changing the parameters in the discriminator neural network *D*_*G*_.

#### Loss Function

The imputation block in MGANITE attempts to impute counterfactual outcomes by extending the loss function of the binary treatment in GANITE (Yoon et al., 2018a) to all types of treatments: binary, categorical or continuous treatments. We define the loss function *V*(*D*_*G*_, *G*) as

$$\begin{array}{l} E_{\left(x,t,m,y_{f})\sim P_{data}(x,t,m,y_{f}\right)}E_{z_{G}\sim u({\left(-1,1\right)}^{K})}\\ \left[M^{T}\log D_{G}\left(X,\tilde{Y},\;T,\;M\right)+{\left(1-M\right)}^{T}\log\left(1-D_{G}\left(X,\tilde{Y},T,\;M\right)\right)\;\right] \end{array}$$

where log is an element-wise operation. The goal of the imputation block is to maximize the counterfactual discriminator *D*_*G*_ and then minimize the counterfactual generator *G*:

(4) $$\begin{array}{l} {\text{min}}_{G}{\text{max}}_{DG}V\left(D_{G},\;G,\;\theta_{d}\right) \end{array}$$

In other words, we train the counterfactual discriminator *D*_*G*_ to maximize the probability of correctly identifying the assigned treatment *M*_η_ and the quantity of the treatment *T*_η_ or *Y*_*f*_(*Y*_η_), and then train the counterfactual generator *G* to minimize the probability of correctly identifying *M*_η_ and *T*_η_. After the imputation block is performed, the counterfactual generator *G* produces the complete dataset $\bar{D}=\left\{x,\;\bar{y}\right\}$. Next, we use the imputed complete dataset $\bar{D}=\left\{X,\;\bar{Y}\right\}$ to generate the distribution of potential outcomes and to estimate the ITE via CGANs which is called the ITE block.

#### ITE Block

The CGANs consist of three parts: generator, discriminator and loss function which are summarized as follows (Yoon et al., 2018a).

##### ITE Generator

Unlike the ITE in GANITE where the ITE generator is a non-linear transform function of only *X* and *Z*_*I*_, the ITE generator *G*_*I*_ in MGANITE is a non-linear transform function of *X, T* and *Z*_*I*_:

(5) $$\begin{array}{l} \hat{Y}=G_{I}\left(X,T,\;Z_{I},\;\theta_{g_{I}}\right) \end{array}$$

where Ŷ is the generated *K*-dimensional vector of potential outcomes, *X* is a feature vector, *T* is a treatment vector, and *Z*_*I*_ is a *K*-dimensional vector of random variables and follows the uniform distribution $Z_{I}\sim u\left({\left(-1,1\right)}^{K}\right)$. The ITE generator attempts to find the transformation Ŷ = *G*_*I*_(*X, T, Z*_*I*_,θ_*g*_*I*__) such that Ŷ~*P*_*Y*|*X, T*_(*y*).

##### ITE Discriminator

Following the CGANs, we define a discriminator *D*_*I*_ as a non-linear classifier with $\left(X,T,Y^{*}=\bar{}Y\right)$ or (X, T, Y^*^ = Ŷ) as input and a scalar that outputs the probability of Y^*^ being from the complete dataset $\bar{}D$.

#### Loss Function

Again, unlike the loss function in GANITE where the decision function is $D_{I}\left(X,Y^{*}\right)$, a decision function in MGANITE is defined as *D*(X, T, Y^*^). The loss function for the ITE block in MGANITE is then defined as

(6) $$\begin{array}{r} V_{I}\left(D_{I},\;G_{I}\right)=E_{X,T\sim P\left(x,T\right)}\\ \left[E_{Y^{*}\sim P_{Y|X,T}}\left(y\right)\left[\log D_{I}\left(X,T,Y^{*}\;\right)\right]+E_{Z_{I}\sim u\left({\left(-1,1\right)}^{K}\right)}\left[\log\left(1-D_{I}X,T,Y^{*}\right)\right]\right] \end{array}$$

where $D_{I}\left(X,{T,\;Y}^{*}\right)$ is the non-linear classifier that determines whether Y^*^ is from the complete dataset $\bar{D}$ or from generator *G*_*I*_.The goal of the ITE block is to maximize the probability of correctly identifying that Y^*^ is from the complete dataset $\bar{D}$ and to minimize the probability of a correct classification. Mathematically, the ITE attempts

(7) $$\begin{array}{l} {\text{min}}_{GI}{\text{max}}_{DI}V_{I}\left(D_{I},\;G_{I}\right) \end{array}$$

The algorithms for numerically solving the optimization problems (4) and (7) are summarized in the Supplementary Note.

The learning parameters for the feedforward neural networks are given below. We set batch size equal to 16. We assumed that the learning rates for the discriminator and generator were 0.0001 and 0.001, respectively. We further assume that the decay rate was 0.1. The learning rate decayed (exponentially) to 10% of the starting learning rate during 70% of the total batches, and stayed at 10% during the last 30% batches. The total number of batches was 1,000,000. Adam Optimizer was used to perform optimization. We assume that 20% of the nodes were dropped randomly during the training process.

### Sparse Techniques for Biomarker Identification

The LASSO (least absolute shrinkage and selection operator) that performs both variable selection and regularization in order to enhance the prediction accuracy and interpretability of the results can be used to select biomarkers for optimal treatment selection (Ali and Aittokallio, 2019). Let $Y_{k}^{i}$ and *X*^(*i*)^denote the estimated effect of the *k*^*th*^ treatment and feature vector of the *i*^*th*^ individual, respectively. Let

$$\begin{array}{l} Y_{T}=\left[\begin{array}{c} Y_{1}^{1}\cdots Y_{K}^{1}\\ \cdots \\ \cdots \\ \cdots \\ Y_{1}^{n}\cdots Y_{K}^{n} \end{array}\right],X=\left[\begin{array}{c} x_{1}^{\left(1\right)}\cdots x_{q}^{\left(1\right)}\\ \cdots \\ \cdots \\ \cdots \\ x_{1}^{\left(n\right)}\cdots x_{q}^{\left(n\right)} \end{array}\right],\;\beta =\left[\begin{array}{c} \beta_{11}\cdots \beta_{1K}\\ \cdots \\ \cdots \\ \cdots \\ \beta_{q1}\cdots \beta_{qK} \end{array}\right] \end{array}$$

The outputs of the neural networks are in general a continuous function even if the potential outcomes are binary. For the convenience of presentation, we assume that the treatment effects are continuous regardless if the potential outcomes are binary, categorical or continuous.

The LASSO estimators for identifying biomarkers that predict treatment effects are given by

(8) $$\begin{array}{l} {\hat{\beta }}_{\lambda }=\text{argmi}{\text{n}}_{\text{β}}||Y_{T}-X\beta |{|}_{F}^{2}+\lambda \overset{q}{\underset{j=1}{\sum }}\overset{K}{\underset{l=1}{\sum }}|\beta_{jl}| \end{array}$$

where ||.||_*F*_ is the Frobenius norm of the matrix. Non-zero elements β_*jl*_≠0 predict treatment effect variation and hence its correspondence $X_{j}={\left[\begin{array}{c} X_{j}^{\left(1\right)}\cdots X_{j}^{\left(n\right)} \end{array}\right]}^{T}$can be used as biomarkers for investigation of the *l*^*th*^ treatment. For the continuous treatment, we define the treatment matrix *T* and its associated coefficient matrix Γ:

$$\begin{array}{l} T=[\begin{array}{c} T_{1}^{\left(1\right)}\cdots T_{K}^{\left(1\right)}\\ \cdots \\ \cdots \\ \cdots \\ T_{1}^{\left(n\right)}\cdots T_{K}^{\left(n\right)} \end{array}],\;\Gamma =\left[\begin{array}{c} \gamma_{11}\cdots \gamma_{1K}\\ \cdots \\ \cdots \\ \cdots \\ \gamma_{K1}\cdots \gamma_{KK} \end{array}\right] \end{array}$$

Equation (8) should be changed to

(9) $$\begin{array}{r} {[\;{\hat{\gamma }}_{\lambda_{1},}\;\hat{\beta }}_{\lambda_{2}}]=\text{argmi}{\text{n}}_{\text{γ},\text{β}}||Y_{T}-T\Gamma -X\beta |{|}_{F}^{2}+\lambda_{1}\overset{K}{\underset{j=1}{\sum }}\overset{K}{\underset{l=1}{\sum }}|\gamma_{jl}|+\;\lambda_{2}\overset{q}{\underset{j=1}{\sum }}\overset{K}{\underset{l=1}{\sum }}|\beta_{jl}| \end{array}$$

where λ_1_, λ_2_ are penalty parameters.

### Biomarker Identification for Optimal Treatment Selection

Consider *K* treatments. Let ${\hat{Y}}^{i}={\left[\begin{array}{c} {\hat{Y}}_{1}^{i}\cdots {\hat{Y}}_{K}^{i} \end{array}\right]}^{T}$ be the *K*-dimensional vector of the estimated potential outcomes for the *i*^*th*^ individual and $z_{i}=\text{argma}{\text{x}}_{1,...,\text{k}}\left\{{\hat{Y}}_{1}^{i},\ldots ,\;{\hat{Y}}_{K}^{i}\;\right\}$ be the index for the optimal potential outcomes of the *i*^*th*^ individual. To select biomarkers for optimal treatment selection, we define the following LASSO:

(10) $$\begin{array}{r} {\hat{Y}}_{z_{i}}^{i}=\overset{q}{\underset{j=1}{\sum }}x_{j}^{\left(i\right)}\alpha_{j}+\lambda \overset{q}{\underset{j=1}{\sum }}|\alpha_{j}|,\;i=1,\ldots ,\;n \end{array}$$

Solving the above categorical LASSO problem, we obtain a set of non-zero coefficients that are denoted as ${\hat{\alpha }}_{l}\neq 0,\;l=1,\ldots ,\;L$. The covariates that correspond to the non-zero coefficients of the LASSO solution are chosen as biomarkers for optimal treatment selection. Again, for the continuous treatment, Equation (10) needs to be changed to

(11) $$\begin{array}{l} {\hat{Y}}_{z_{i}}^{i}=\overset{K}{\underset{l=1}{\sum }}T_{l}^{\left(i\right)}\delta_{l}+\overset{q}{\underset{j=1}{\sum }}x_{j}^{\left(i\right)}\alpha_{j}+\lambda_{1}\overset{K}{\underset{l=1}{\sum }}|\delta_{l}|+\lambda_{2}\overset{q}{\underset{j=1}{\sum }}|\alpha_{j}|,i=1,\ldots ,\;n. \end{array}$$

### Data Collection

The proposed MGANITE was applied to 256 newly diagnosed acute myeloid leukemia (AML) patients, treated with high dose ara-C (HDAC), Idarubicin (IDA), and HDAC+IDA at M. D. Anderson Cancer Center. There were 212 valid samples and 85 useable features (14 discrete and 71 continuous), including 51 total and phosphoprotein from several biological processes such as apoptosis, cell-cycle, and signal transduction pathways (Kornblau et al., 2009). Among the 212 valid samples, 37 were treated with HDAC, 9 were treated with IDA and 54 were treated with HDAC+IDA, and 112 were treated with other drugs. Data were downloaded from the M. D. Anderson Cancer Center database (http://bioinformatics.mdanderson.org/Supplements/Kornblau-AML-RPPA/aml-rppa.xls) and (https://pubmed.ncbi.nlm.nih.gov/18840713/).

Prediction accuracy was defined as the proportions of correctly predicted potential outcomes. The false positive rate was defined as the proportion of individuals who were wrongly classified as having a positive treatment response. Discriminator accuracy is defined as the proportion of correctly classified real or fake samples. Replication error is defined as cross entropy −*y*_*f*_logŷ_*f*_ where ${ŷ}_{f}=G\left(x,t,t_{{}^{*}},\;y_{f},z_{G},\;\theta_{g}\right),\;t=t_{{}^{*}}$ and separate distance is defined as

$$\begin{array}{l} \frac{1}{n}\overset{n}{\underset{i=1}{\sum }}|y_{if}-{ŷ}_{if}| \end{array}$$

where ${ŷ}_{if}=G\left(x,t,t_{{}^{*}},\;y_{f},z_{G},\;\theta_{g}\right),\;t\neq t_{{}^{*}}$.

## Results

### Simulations

We first examine the performance of MGANITE in estimating the ITE of binary treatment using simulations. A synthetic dataset is generated as follows. A total of 10,000 individuals with 30-dimentinal feature vectors follow the normal distributions *N*(0, *I*). Let

$$\begin{array}{l} {ŷ}_{i}^{0}=0.05+0.4x_{i1}^{2}+0.25x_{i2}+n_{i0},n_{i0}\sim N\left(0,\;0.05\right) \end{array}$$

and

$$\begin{array}{l} {ŷ}_{i}^{1}=0.15+{0.5x}_{i1}^{2}+0.25x_{i1}x_{i2}+0.25x_{i2}+n_{i1}\\ i=1,2,\ldots ,\;10,000,n_{i1}\sim N\left(0,\;0.05\right), \end{array}$$

where *i* is a sample index.

Then, the potential outcomes are generated as

$$y_{i}^{0}=\{\begin{array}{c} 1\;{\hat{y}}_{i}^{0}\geq 0.5\\ 0\;{\hat{y}}_{i}^{0}<0.5 \end{array}\text{ and }y_{i}^{1}=\{\begin{array}{c} 1\;{\hat{y}}_{i}^{1}\geq 0.5\\ 0\;{\hat{y}}_{i}^{1}<0.5 \end{array}$$

Treatment is assigned by the Bernoulli distribution:

$$\begin{array}{l} M=T|X\sim Bern\left(sigmoid\left(W_{t}^{T}X+n_{t}\;\right)\right) \end{array}$$

where *t* is a treatment index, $W_{t}^{T}\sim u{\left(-0.1,0.1\right)}^{30\times 1}$, *n*_*t*_~*N*(0, 0.1), and Bern represents the Bernoulli distribution. When one sample has only one treatment assigned, then *t* = *i*.

Treatment effect can take three values 1, 0, and −1. In other words,

$$\xi_{i}=\{\begin{array}{ll} 1 & y_{i}^{1}=1,\;y_{i}^{0}=0\\ \begin{array}{c} 0\;y_{i}^{1}=1,\;y_{i}^{0}=1 \end{array}\;or & y_{i}^{1}=0,\;y_{i}^{0}=0\\ -1 & y_{i}^{1}=0,\;y_{i}^{0}=1 \end{array}$$

**Figure 2:**
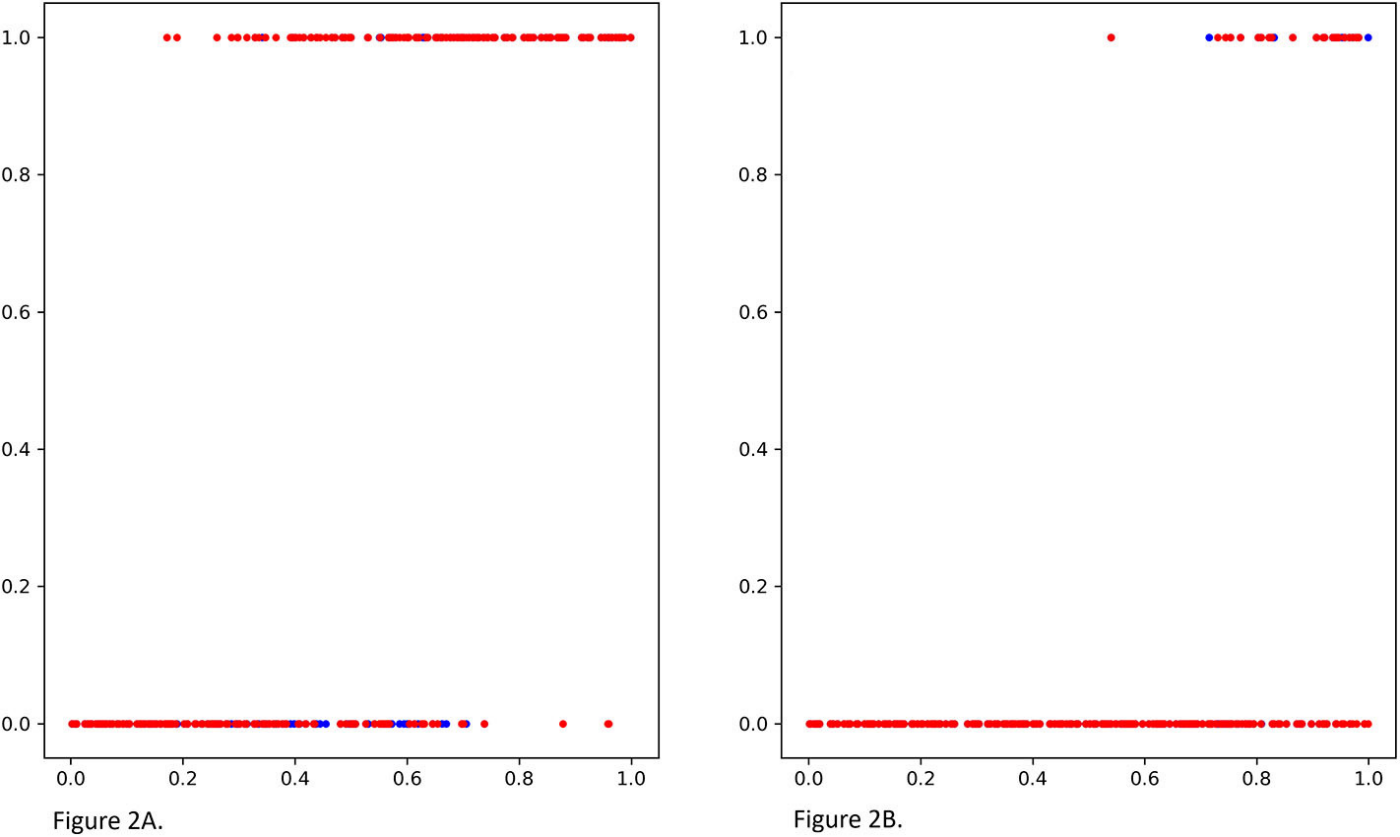
**(A)** The true potential outcomes with treatment *Y*^1^ and estimated potential outcomes ŷ^1^ using MGANITE, where the *x* axis denoted a value of covariate *X*_1_, the *y* axis denoted the potential outcome, a blue color dot represented the true outcome *Y*^1^ and a red color dot represented the estimated outcomes ŷ^1^. **(B)** The true potential outcomes without treatment *Y*^0^and estimated potential outcomes ŷ^0^ using MGANITE, where the *x* axis denoted a value of covariate *X*_1_, the *y* axis denoted the potential outcome, a blue color dot represented the true outcome *Y*^0^ and a red color dot represented the estimated outcomes ŷ^0^.

We compare MGANITE with linear regression (LR) (Makar et al., 2019), logistic regression (LogR) (Emmert-Streib and Dehmer, 2019; Makar et al., 2019), support vector machine (SVM) (Makar et al., 2019), *k*- nearest neighbor (k-NN) (Crump et al., 2008), Bayesian linear regression (BLR) (Johansson et al., 2016), causal forest (CForest) ( Wager and Athey, 2015), and random forest classification [RF (C)] (Breiman, 2001). We use six methods: MGANITE, LR, LogR, SVM, kNN, and RF (C) to estimate the counterfactual potential outcomes and calculate the mean square error (MSE) between the estimated treatment effect and the true treatment effect, standard deviation (STD) and prediction accuracy. Table 1 presents MSE, STD, and prediction accuracy of six methods to fit the generated data. We observe that MGANITE more accurately estimate the potential outcomes than the other five state-of-the-art methods. Figure 2 presents the true counterfactuals and estimates counterfactuals using MGANITE. We observe that MGANITE reaches remarkably high accuracy for estimating counterfactuals.

**Table 1:** Performance of six methods for estimating the potential outcomes.

**Methods**	**MSE**	**STD**	**Accuracy**
MGANITE	0.062	0.235	0.938
LR	0.104	0.305	0.896
LogR	0.120	0.325	0.880
SVM	0.126	0.332	0.874
KNN	0.148	0.355	0.852
RF (C)	0.098	0.297	0.902

The treatment effect estimation of eight methods [MGANITE, LR, LogR, SVM, KNN (5,10), BLR, RF (C), RF (R)] are summarized in Table 2. Table 2 shows that MGANITE has the highest accuracy of estimation of all treatment effects: average treatment effect (ATE), average treatment effects on the treated (ATT), and average treatment effect on the control (ATC), followed by RF (R) or RF (C). We observe that the estimations of ATE using all methods are inflated. The inflation rates of ATE using MGANITE and RF (C) are 3.9 and 7.9%, respectively. The SVM reaches the inflation rate of the estimation of ATE as high as 29.8%. All inflation rates of estimation of ATE using LR, LogR, SVM, KNN, and BLR are very high. The simulations also show that the false positive rates using MGANITE, LR, LogR, SVC, KNN (5), KNN (10), BLR, RF (R), and RF (C) are 3.9, 24.7, 28.1, 29.8, 28/1, 19.7, 25.3, 9, and 8.4%, respectively. The results show that false positive rates of LR, LogR, SVM, KNN, and BLR for prediction of positive treatment response are too high to be applied to treatment selection. Even RF (R) reaches the false positive rate as high as 8.4%. Table 2 also shows that the number of individuals that show positive treatment effects increases while the number of individuals that show no treatment effect decreases from ground truth.

**Table 2:** Treatment effects estimated for simulation data using nine methods.

**Methods**	**ATT**	**ATC**	**ATE**	**ITE = −1**	**ITE = 0**	**ITE = 1**
Ground truth	0.391	0.321	0.356	0	322	178
MGANITE	0.399	0.341	0.37	0	315	185
LR	0.52	0.369	0.444	0	278	222
LogR	0.52	0.393	0.456	0	272	228
SVM	0.524	0.401	0.462	0	269	231
KNN (5)	0.508	0.401	0.454	1	271	228
KNN (10)	0.524	0.325	0.424	1	286	213
BLR	0.524	0.369	0.446	0	277	223
RF (C)	0.452	0.325	0.388	0	306	194
RF (R)	0.431	0.337	0.384	1	306	193

Next we examine the performance of MGANITE in estimating the ITE of continuous treatment using simulations. A synthetic dataset is generated as follows.

1. Draw the covariate variable *X* from the standard normal distribution for 10,000 individuals.
2. The treatment *T* is exponentially distributed as *P*(*t*) = *e*^−(*t*−1)^, *t* ≥ 1. Define *g*(*t*) = 0.1*t*^2^.
3. Define a non-linear function $f\left(x\right)=\frac{1}{2+exp\left(-20\left(x-\frac{1}{3}\;\right)\right)}$.
4. Define $y_{i}^{0}=0.3+f\left(x\right)+n_{i}^{0},\;i=1,..,\;10,000$, where $n_{i}^{0}$ is a randomly sampled noise variable from a normal distribution *N*(0, 0.01).
5. Define $y_{i}^{1}=0.3+f\left(x\right)+g\left(t\right)+n_{i}^{1},\;i=1,\ldots ,\;10,000$, where $n_{i}^{1}$ is a randomly sampled noise variable from a normal distribution *N*(0, 0.01).
6. Treatment assignment indicator variable *M*_*i*_ is drawn from a Bernoulli distribution with *P* = 0.5 for each subject.

**Figure 3:**
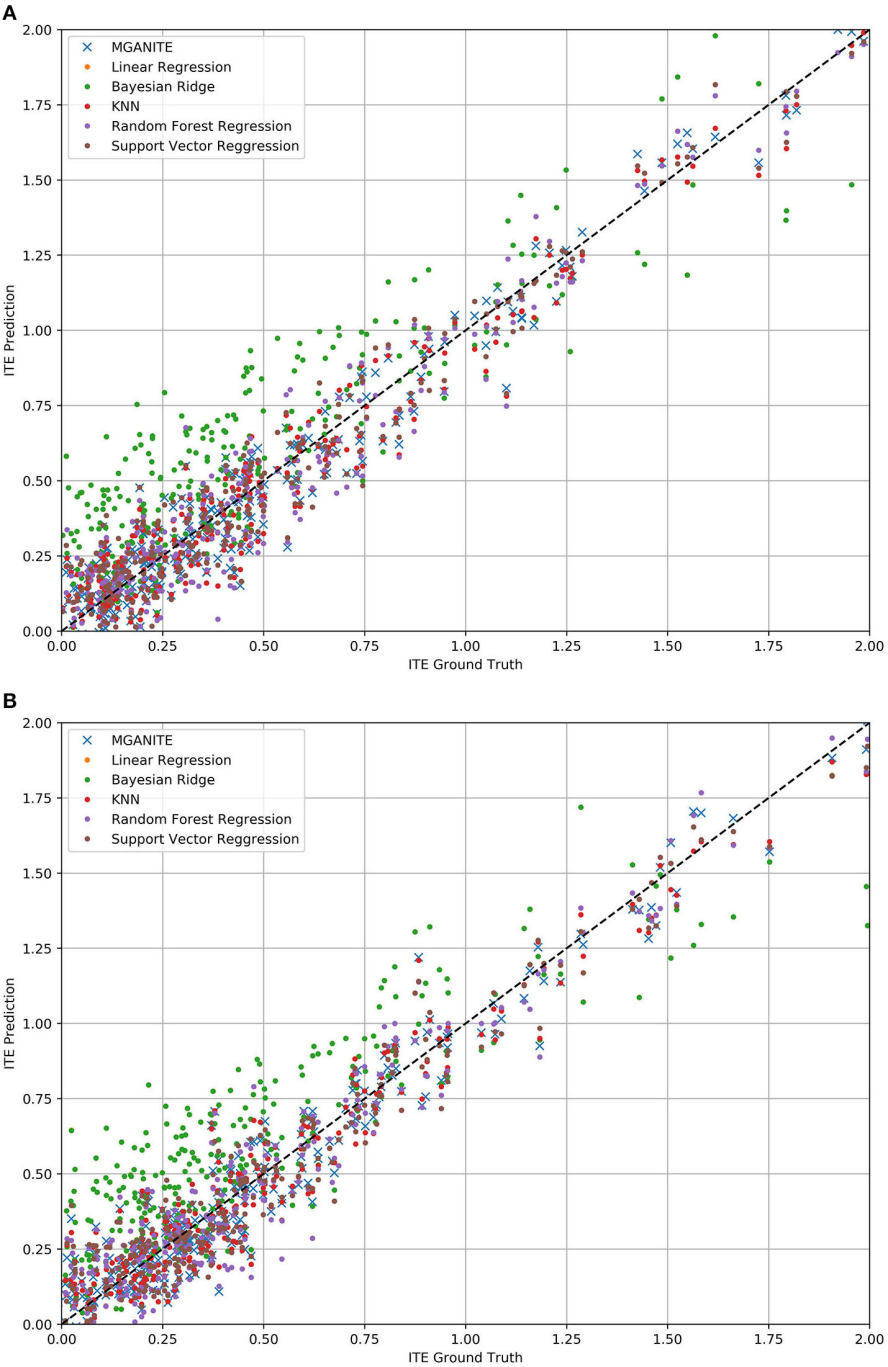
**(A)** True ITE and estimated ITE for in-sample data using six methods: MGANITE, LR, KNN, BLR, RF (R), and SVM, where MGANTE was denoted by a green cross point, LR was denoted by an orange point, KNN was denoted by a green point, BLR was denoted by a red point, RF (R) was denoted by a purple point and SVM was denoted by a dark red point, the *x* axis denoted the true ITE and the *y* axis denoted the estimated ITE. **(B)** True ITE and estimated ITE for out-of-sample data using six methods: MGANITE, LR, KNN, BLR, RF (R), and SVM, where MGANTE was denoted by a green cross point, LR was denoted by a orange point, KNN was denoted by a green point, BLR was denoted by a red point, RF (R) was denoted by a purple point and SVM was denoted by a dark red point, the *x* axis denoted the true ITE and the *y* axis denoted the estimated ITE.

The mean square errors (MSE) for MGANITE, Linear Regression, KNN, Bayesian ridge regression, RF (R), and SVM regression are 0.011004916, 0.08500695, 0.012520364, 0.085007192, 0.014281599, 0.013962992, respectively. Figures 3A,B plot the true ITE and estimated ITE for in-samples and out-of-samples data, using six methods: MGANITE, LR, KNN, BLR, RF (R), and SVM, respectively, where a dash straight line indicates that the true ITE and the estimated ITE are equal. We observe from Figures 3A,B that many green cross points for both in-sample and out-of-sample data are much closer to the dash straight line than other types of points. This shows that the estimated ITE points using MGANITE are much closer to the true ITE point than using the other five methods. In other words, the estimator of the ITE using MGANITE is more accurate than that of using the other five methods. The results clearly demonstrate that MGANITE outperforms the 5 other state-of-the-art treatment effect estimation methods.

**Figure 4:**
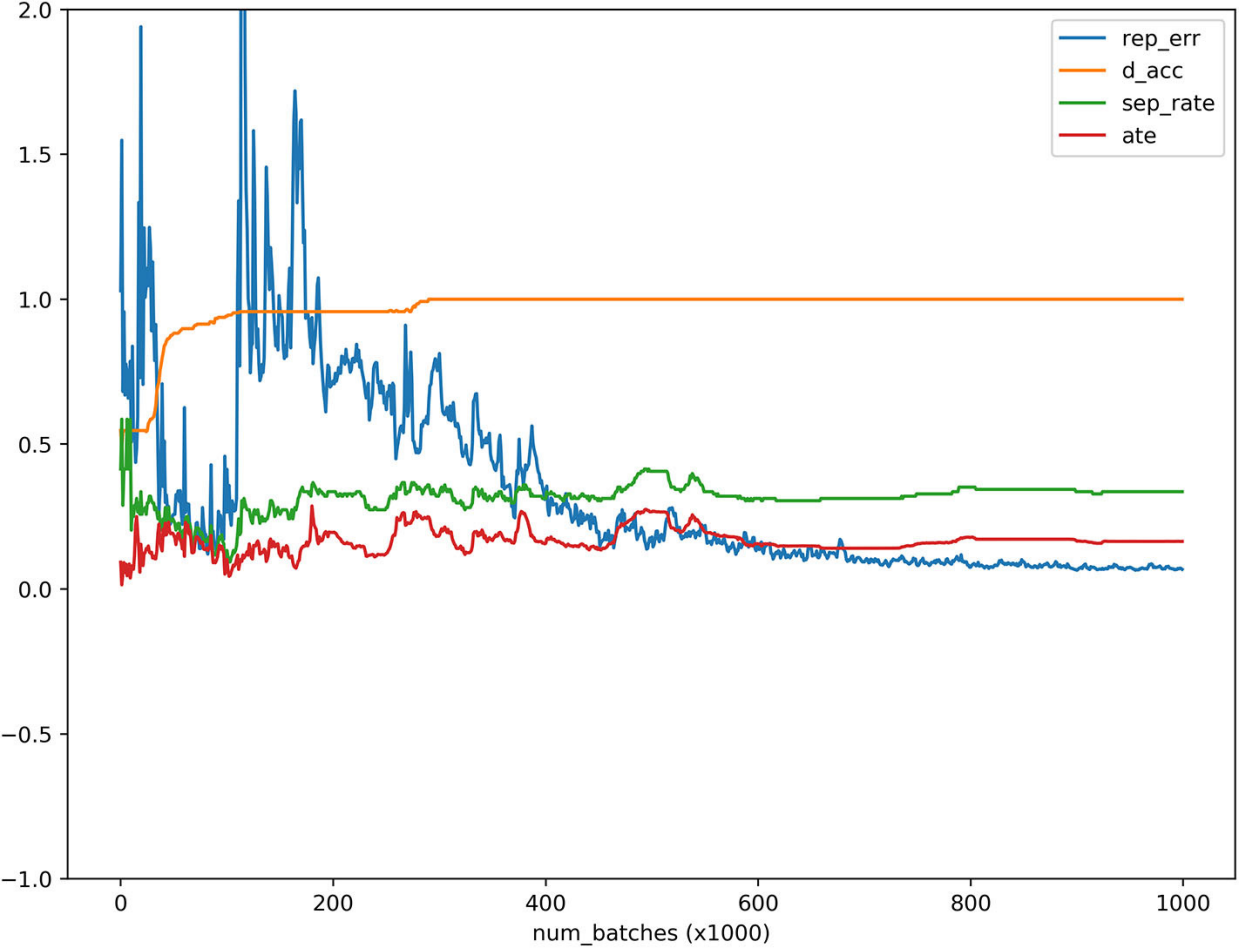
ATE, discriminator accuracy, replication error and separate distance curves as a function of the number of batches where the *x* axis denoted the number of batches, the *y* axis denoted values of the ATE, discriminator accuracy, replication error, and separation distance for ATE, discriminator, replication, and separation curves, respectively, red, orange, blue and green curves were ATE, discriminator, replication and separation curves, respectively.

To further evaluate the performance of MGANITE, we provide Figure 4 that plots the receiver operating characteristic (ROC) curve for evaluation of the ability of MGANITE to predict potential outcomes of treatment. Our calculation shows that area under the ROC curve (AUC) for MGANITE reaches 0.98, which is a very high value. The ROC curve and AUC value demonstrate that the power of MGANITE for prediction of the potential outcomes of the treatments is very high.

### Real Data Analysis

**Figure 5:**
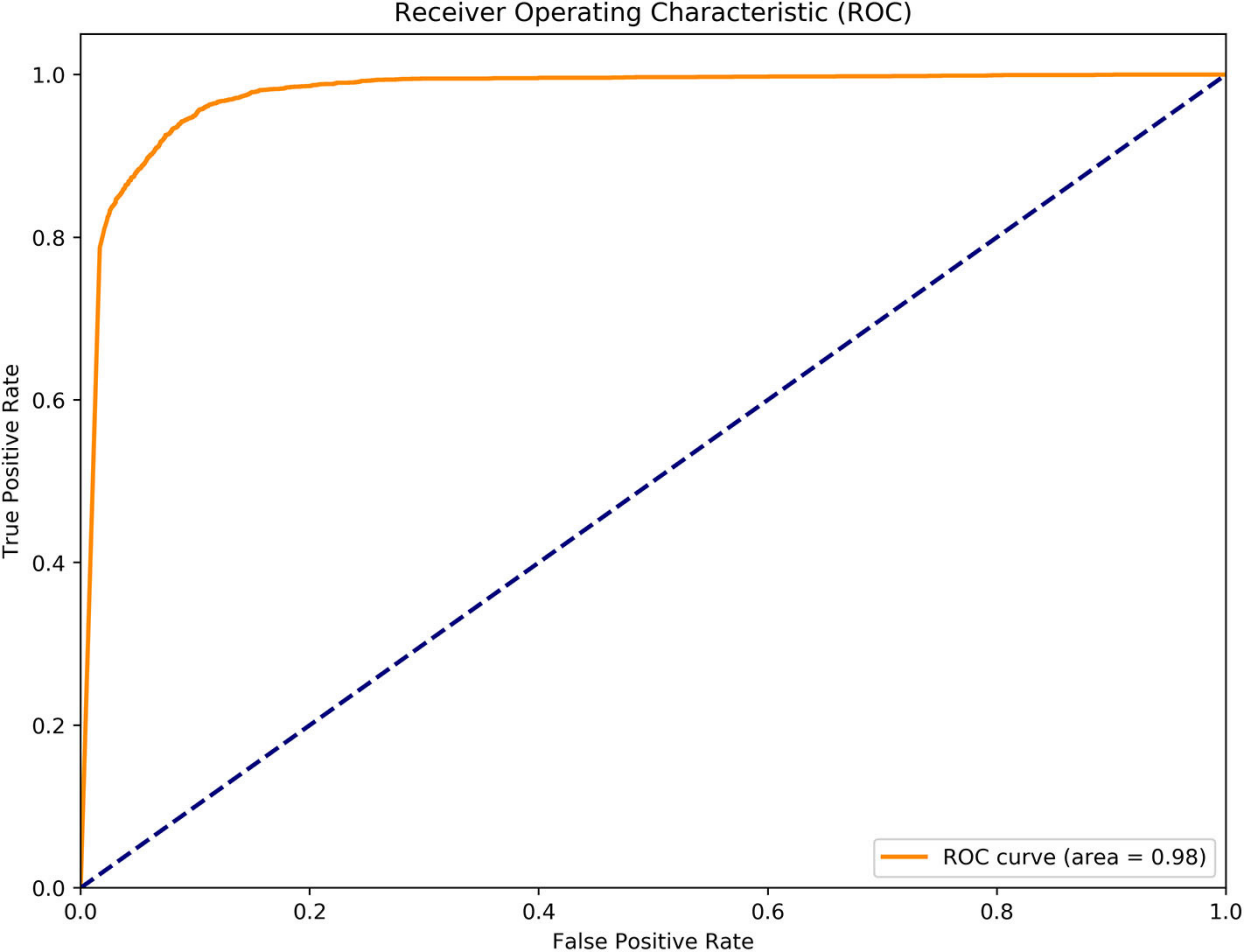
Receiver operating characteristic (ROC) curve for evaluation of performance of MGANITE.

MGANITE is applied to 256 newly diagnosed acute myeloid leukemia (AML) patients from the clinical trial dataset (Kornblau et al., 2009). We first present the results of treatment using HDAC, HDAC+IDA (101) vs. all other drugs (111). A key issue for MGANITE is how to train MGANITE. To track the training process of MGANITE, we present Figure 5 that shows ATE, discriminator accuracy, replication error, and separate distance curves as a function of the number of batches.

We observe from Figure 5 that discriminator accuracy converges to 1, replication error converges to zero, separation distance converges to a constant, and ATE converges to a stable value. Figure 4 demonstrates that MGANITE is trained very well.

Next we compared the treatment effect estimations using nine methods: MGANITE, LR, LogR, SVM, KNN (5), KNN (10), BLR, RF (C), and RF (R) where 5 and 10 are the number of neighbors. Treatment with HDAC or HDAC+IDA, and 85 protein expressions and other geographical variables are used as covariates. The response status (response or no response) is used as the outcome.

**Figure 6:**
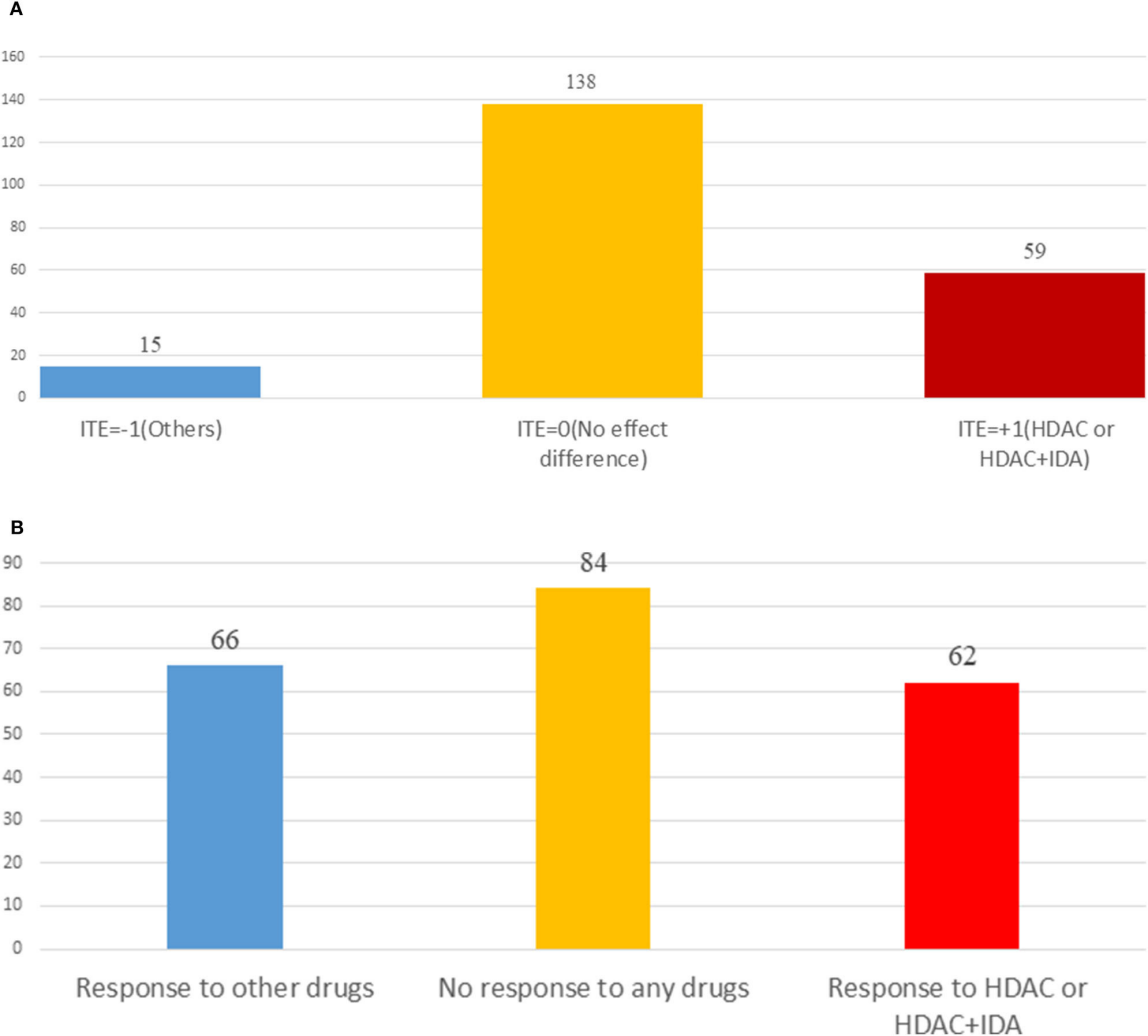
**(A)** Histogram of estimated drug treatment effect using MGANITE, where the *x* axis denoted the value of ITE and the *y* axis denoted the number of patients, *ITE* = +1 denoted the ITE of patients treated with HDAC or HDAC+IDA, *ITE* = −1 denoted the ITE of patients treated with other drugs, and *ITE* = 0 denoted the ITE of two groups of patients: one group of the patients treated with HDAC or HDAC+IDA and another group of the patients treated with other drugs. **(B)** Histogram of observed drug treatment response where the *x* axis indicated three scenarios as described in **(B)** and the *y* axis denoted the number of patients, the right side in the **(B)** denoted the number of patients only responding to the HDAC or HDAC+IDA, the middle denoted the number of the patients that responds to both (HDAC or HDAC+IDA) and other drugs or did not respond to both (HDAC or HDAC+IDA) and other drugs, and the left side denoted the number of patients only responding to the other drugs.

Table 3 summarizes results of the estimation of HDAC treatment effect using MGANITE and other eight methods where individuals with HDAC or HDAC+IDA are taken as the treated population and individuals with other drugs are taken as the control population. Comparison of treatment effect estimation algorithms on real data analysis is not easy because of the lack of ground truth treatment effects and small sample sizes. In general, using MGANITE, we observe that the majority of individuals who are treated by other drugs do not show any response and that 65% of the individuals who are treated by HDAC or HDAC+IDA respond. Only 13.5% of individuals who are treated by other drugs respond. To illustrate the difference between the estimated treatment effect and treatment response, we present Figure 6 that shows the histogram of the estimated effects of the treatments HDAC or HDAC+IDA vs. other drugs using MGANITE (Figure 6A), and observe the number of responses of the individuals in the population who are treated with HDAC or HDAC+IDA vs. other drugs (Figure 6B). ITE is calculated based on both the factual and counterfactual. We observe that *ITE* = 0 consists of two scenarios: (1) no response of the patients to any drugs and (2) response of the patients to both HDAC or HDAC+IDA, and other drugs. A proportion of the patients with response to HDAC or HDAC+IDA on the right side of Figure 6B and the patient with response to other drugs on the left side of Figure 6B has *ITE* = 0. The observed response of the patients to one drug does not imply that these patients would not respond to other drugs. However, *ITE* = 1 or *ITE* = 0 implies that the patients respond to only one type of drug. To further compare the performance of MGANITE and other methods for evaluation of ITE, we split a given data set into an in-sample dataset (190 samples), used for the initial parameter estimation and model selection, and an out-of-sample dataset (22 samples), used to evaluate performance of ITE estimation. The results are summarized in Table 4. We observe that the difference in the estimated ATT, ATC, ATE and proportions of the ITE between in-samples and out-of-samples using MGANITE are much smaller than using other methods. This shows that the ITE estimation using MGANITE is more robust than using other methods. We calculate the Kullback-Leibler (K-L) divergence between the distributions of the ITE using in-sample and out-of-samples, and using nine methods. The results are summarized in Table 5. Table 5 shows that K-L divergence using MGANITE is much smaller than that using other methods, which implies that MGANITE is more robust than the other eight methods.

**Table 3:** Treatment effects estimated for AML dataset using nine methods.

**Methods**	**ATT**	**ATC**	**ATE**	**Number of individuals with positive treatment effect**		
				**HDAC and**	**No**	**Other**
				**HDAC+IDA**	**difference**	**drugs**
CGANs	0.011	0.356	0.208	59	138	15
LR	0.033	0.207	0.107	62	112	38
LogR	0.083	0.209	0.137	63	115	34
SVM	0.112	0.165	0.135	65	130	17
KNN (5)	0.248	−0.011	0.137	55	131	26
KNN (10)	0.314	0.066	0.208	62	132	18
BLR	0.129	0.139	0.133	57	136	19
RF (C)	0.157	0.286	0.212	70	117	25
RF (R)	0.052	0.099	0.072	37	155	20

**Table 4:** Treatment effects estimated for AML dataset using nine methods.

**Method**	**ATT**	**ATC**	**ATE**	**ITE = −1**	**ITE = 0**	**ITE = 1**
				**Proportion**		
**In-sample**						
MCGAN	0.3152	0.2733	0.2911	0.0842	0.5474	0.3684
LR	0.1077	−0.021	0.0339	0.2474	0.4789	0.2737
BLR	0.0843	0.0817	0.0828	0.1158	0.6684	0.2158
KNN (5)	−0.0247	0.1743	0.0895	0.1474	0.6158	0.2368
KNN (10)	0.0494	0.1835	0.1263	0.1211	0.6316	0.2474
RF (C)	0.2099	0.0826	0.1368	0.1421	0.5789	0.2789
RF (R)	0.0852	0.0459	0.0626	0.1316	0.6737	0.1947
LogR	0.1358	0.1193	0.1263	0.1579	0.5579	0.2842
SVM	0.1081	0.0571	0.0788	0.1158	0.6263	0.2579
**Out-of-sample**						
MCGAN	0.2000	0.3266	0.2691	0.0455	0.6364	0.3182
LR	0.4974	0.1222	0.2928	0.0909	0.5000	0.4091
BLR	0.3470	0.3129	0.3284	0.0000	0.5909	0.4091
KNN (5)	0.3000	0.5000	0.4091	0.0455	0.5000	0.4545
KNN (10)	0.2000	0.5000	0.3636	0.0000	0.6364	0.3636
RF (C)	0.0000	0.3333	0.1818	0.0455	0.7273	0.2273
RF (R)	0.2600	0.3583	0.3136	0.0000	0.7273	0.2727
LogR	0.6000	0.4167	0.5000	0.0000	0.5000	0.5000
SVM	0.3502	0.2823	0.3132	0.0000	0.5455	0.4545

**Table 5:** K-L divergence between the distribution of ITEs using in-samples and out-of-samples.

**Methods**	**Kullback–Leibler divergence**
MGANITE	0.00920
LR	0.04123
BLR	0.08201
KNN (5)	0.06024
KNN (10)	0.06293
RF (C)	0.02932
RF (R)	0.06407
LogR	0.09887
SVM	0.07913

LASSO is used to identify biomarkers for prediction of treatment effect and treatment selection. Table 6 lists the top 30 biomarkers identified by LASSO. All top 30 biomarkers explain 36.82% of the variation of HDAC or HDAC+IDA treatment effect. The top Gene *GSK3* accounts for 4.4% of the explanation of treatment effect variation.

**Table 6:** Top ranking variables for explanation of treatment effect variation.

**Gene name**	**R-square (single)**	**R-square (accumulated)**	**Gene name**	**R-square (single)**	**R-square (accumulated)**
GSK3	0.0440	0.0440	CD33	0.0134	0.1984
BILIRUBIN	0.0411	0.0790	TP53	0.0118	0.2376
DIABLO	0.0370	0.1266	STAT3	0.0085	0.2415
SRC	0.0333	0.1329	BIRC5	0.0071	0.2421
MEK	0.0282	0.1373	BAX	0.0070	0.2446
AKT.p308	0.0244	0.1405	DJI	0.0061	0.2591
Age_at_Dx	0.0226	0.1488	CREATININE	0.0057	0.2627
PRIOR_XRT	0.0202	0.1776	BAD	0.0052	0.2646
PSMC4	0.0196	0.1844	ACTB	0.0052	0.2816
PB_Blast	0.0181	0.1858	WBC	0.0045	0.2922
BM_Blast	0.0167	0.1878	PRIOR_MAL	0.0042	0.3190
CD20	0.0167	0.1883	FIBRINOGEN	0.0038	0.3213
NRP1	0.0147	0.1914	STAT6	0.0033	0.3383
TP38.p	0.0143	0.1954	CD13	0.0033	0.3409
PSMC4	0.0135	0.1971	PTEN	0.0030	0.3682

Garson's algorithm (Garson, 1991; Siu, 2017; Zhang et al., 2018) that describes the relative magnitude of the importance of input variables (biomarkers) in its connection with outcome variables (ITE) of the neural network can also be used to identify biomarkers for predicting the ITE. The top 30 biomarkers identified by the Garson algorithm are listed in Supplementary Table 1 where the relative contribution of each biomarker to the ITE variation and cumulative contribution of biomarkers to the ITE variation are also listed in Supplementary Table 1. The correlation coefficient between the importance ranking of the markers using the Garson algorithm and LASSO is only −0.05.

Next, we study the joint estimation of effects of the multiple treatments. The number of individuals that are treated with HDAC, HDAC+IDA, and other drugs are 37, 54, and 121, respectively. The widely used treatment estimation methods with multiple treatments are simultaneous estimations of the effects of pairwise treatments. We estimate the effects of the pairwise treatments HDAC vs. HDAC+IDA, HDAC vs. other drugs, and HDAC+IDA vs. other drugs. The results are summarized in Table 7. Pairwise comparisons listed in Table 7 does not present the results of the treatment compared with a placebo (without using any drugs). We compare the effect of one treatment with another treatment. Specifically, we make pairwise comparisons: HDAC vs. other drugs, HDAC+IDA vs. other drugs, and HDAC+IDA vs. HDAC. The average treatment effects (ATE) of these three pairwise treatments: HDAC vs. other drugs, HDAC+IDA vs. other drugs, and HDAC+IDA vs. HDAC using MGANITE, are 0.1001, 0.2311 and 0.1310, respectively. This demonstrates that on the average, the effect of the HDAC+IDA is the largest among the three treatments: HDAC+IDA, HDAC, and other drugs, followed by the treatment HDAC. In other words, the treatment HDAC is better than other drugs, in turn, the combination of HDAC and IDA is better than HDAC. It is also noted that the effect of HDAC+IDA vs. other drugs—effect of HDAC vs. other drugs = 0.2311–0.1001 = 0.1310 = effect of HDAC+IDA vs. HDAC.

**Table 7:** Multiple treatment effects estimated for AML dataset using nine methods.

	**ATE**	**Number of individuals with treatment effect**		
**Method**	**HDAC vs. other**	**HDAC**	**No difference**	**Other**
MGANITE	0.1001	59	115	38
LR	0.1149	58	122	32
LogR	0.0896	54	123	35
SVM	0.1463	59	140	13
KNN (5)	0.1887	62	128	22
KNN (10)	0.3538	80	127	5
BLR	0.0860	45	138	29
RF (C)	0.2264	73	114	25
RF (R)	0.2127	58	145	9
**Method**	**HDAC+IDA vs. other**	**HDAC+IDA**	**No difference**	**Other**
MGANITE	0.2311	79	103	30
LR	0.0965	59	115	38
LogR	0.2123	56	115	41
SVM	0.2453	52	138	22
KNN (5)	0.1012	62	133	17
KNN (10)	0.1604	63	138	11
BLR	0.1307	49	137	26
RF (C)	0.0708	70	106	36
RF (R)	0.0835	43	155	14
**Method**	**HDAC+IDA vs. HDAC**	**HDAC+IDA**	**No difference**	**HDAC**
MGANITE	0.1310	52	136	24
LR	−0.0184	36	130	46
LogR	−0.0189	45	118	49
SVM	−0.0628	9	181	22
KNN (5)	0.0236	34	149	29
KNN (10)	−0.1085	11	167	34
BLR	0.0152	40	139	33
RF (C)	−0.0660	31	136	45
RF (R)	−0.0821	8	184	20

However, using LR, LogR, SVM, RF (C), and RF (R), we observe that HDAC is the best treatment. This conclusion violates the biological interpretation. We explain the reasons that causes this incorrect conclusion as follows. The traditional methods for treatment estimation are mainly based on the population average of the treatment responses. The number of observed responses and no responses of the individuals treated with other drugs is 66 and 55, respectively. The average response rate of the other drugs is 0.545. The number of observed responses and no responses of the individuals treated with HDAC is 29 and 8, respectively. The average response rate for HDAC is 0.784. The number of observed response and no response of individuals treated with HDAC + IDA is 33 and 21, respectively. The average response rate for HDAC +IDA is 0.611. Therefore, estimators of ATE for the treatment of HDAC vs. other drugs using LR, LogR, SVM, RF (C), and RF (R) are higher than the estimators of ATE for the HDAC + IDA treatment. However, the individuals treated with HDAC+IDA usually do not respond to HDAC treatment. Therefore, the number of individuals with no response should be adjusted to 62. After adjustment, the response rate of HDAC is changed to 0.319. Therefore, after adjustment, the ATE of HDAC vs. other drugs is smaller than the ATE for HDAC +IDA. Then, the estimators of the pair-wise treatments using MGANITE are consistent with the treatment responses after the data are adjusted. This example shows that these traditional methods that are designed for single treatment effect estimation should be modified when they are applied to multiple treatment effect estimation.

**Figure 7:**
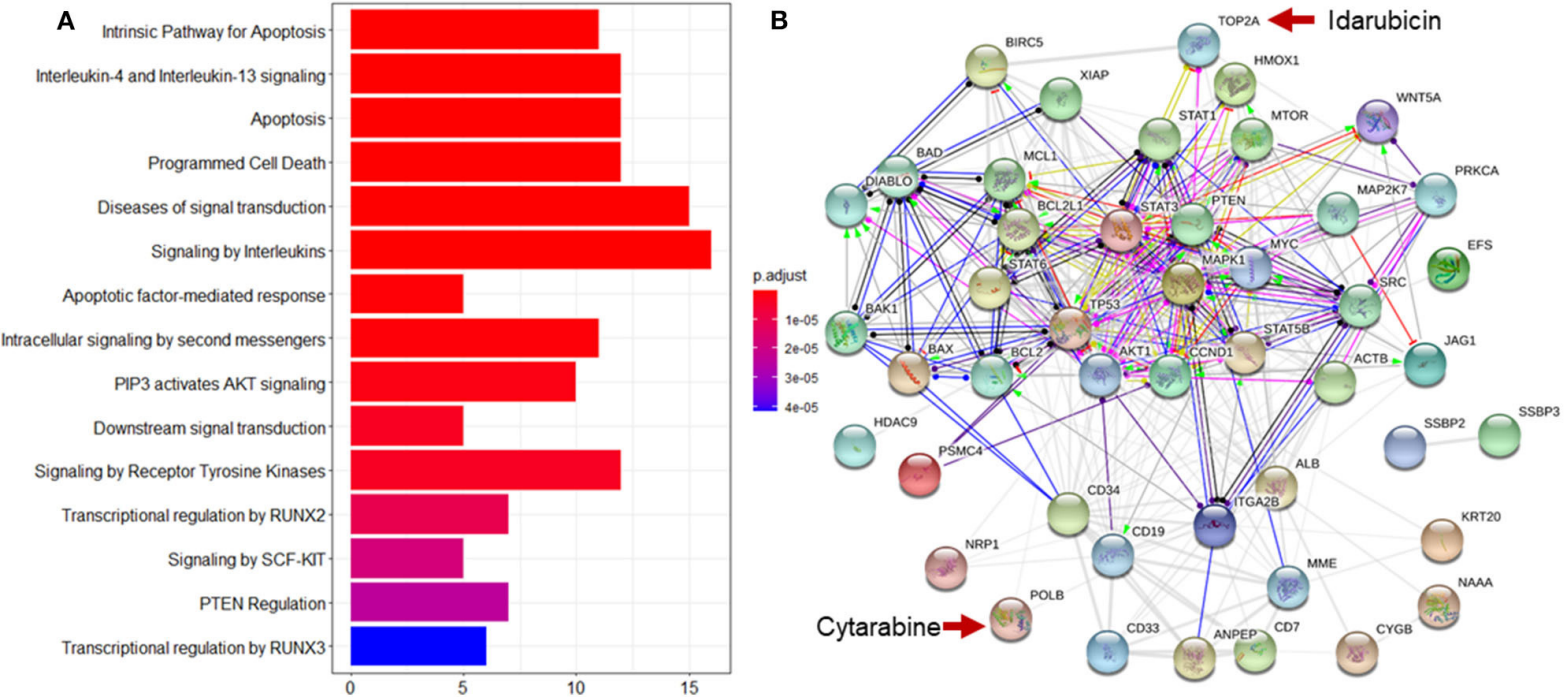
Reactome pathway analysis and protein-protein interaction (PPI) network analysis to top ranking biomarkers for explanation of treatment effect variation. **(A**) Enrichment analysis to the top 44 ranking biomarkers for explanation of treatment effect variation with the Reactome pathway database by hypergeometric test to assess whether the number of identified biomarkers associated with the Reactome pathway was over-represented more than expected. The original *P*-value from the hypergeometric test was then adjusted by FDR for multiple test correction. The top 15 most significantly enriched pathways was shown. **(B)** PPI network analysis was performed by String 11.0 to show the protein-protein interaction among top ranking biomarkers. We found that these proteins were highly interacted which was consistent with pathway enrichment analysis (PPI enrichment *P*-value is 1.0e-16).

Enrichment analysis to top ranking variables for explanation of treatment effect variation is performed by the hypergeometric test via the Reactome Pathway Database (RPD) (Jassal et al., 2020) to assess whether the number of identified biomarkers associated with the Reactome pathway is over-represented more than expected. The original *P*-value from the hypergeometric test is then adjusted by FDR for multiple test correction. We find that top ranking biomarkers for the explanation of treatment effect variation are enriched in multiple cancer related pathways (Figure 7A), including the intrinsic pathway for apoptosis (R-HSA-109606, *P* = 2.86 × 10^−14^), Signaling by Interleukins (R-HSA-449147, *P* = 2.86 × 10^−14^), Programmed Cell Death (R-HSA-5357801, *P* = 9.7 × 10^−11^), PIP3 activates AKT signaling (R-HSA-1257604, *P* = 2.98 × 10^−8^), RUNX3 regulates WNT signaling (R-HSA-8951430, *P* = 1.03 × 10^−5^), and RNA Polymerase II Transcription (R-HSA-73857, *P* = 9.4 × 10^−5^). In addition, we find that the drug target of idarubicin (TOP2A) and Cytarabine (POLB) form a significant protein-protein interaction network (*P* < 1.0 × 10^−16^) (Szklarczyk et al., 2019), indicating that the predictive biomarkers work as the direct interactive proteins of cancer drug targets (Figure 7B).

## Discussion

In this paper, we present MGANITE coupled with sparse techniques as a framework to estimate the ITEs and select the optimal treatments. We demonstrate that the proposed MGANITE has several remarkable features.

First, MGANITE extends GANITE from binary treatment to all types of treatments: binary, categorical, and continuous treatments. We show that MGANITE has a much higher accuracy for estimation of ITE than other state-of-the-art methods.

Second, in-sample and out-of-sample analysis show that the K-L divergence between the distributions of ITE for in-sample and out-of-samples for MGANITE is much smaller than that of other methods, which implies that MGANITE is more robust than other state-of-the art methods.

Third, unlike many popular methods that are usually used to estimate the average effect of the single treatment, MGANITE not only can estimate the ITE of a single treatment, but also can accurately and jointly estimate the ITE of multiple treatments. We also show that the results of the joint estimation of multiple treatments using other classical methods are inconsistent and might violate the biological interpretation.

Fourth, precision oncology is the identification of the right treatment for the right patient. The essential aim is to discover biomarkers that can accurately predict individual treatment effect for each individual. Our results show that MGANITE with sparse techniques can identify a set of biomarkers with significant biological features. The following identified biomarkers are such typical examples.

*GSK3* is a kinase so adaptable that it has been recruited evolutionarily to phosphorylate over 100 substrates, and can regulate numerous cellular functions (Beurel et al., 2015). *GSK3* phosphorylates HDAC3 and promotes its activity, including the neurotoxic activity of HDAC3 (Bardai and D'Mello, 2011). *GSK3* also phosphorylates HDAC6 to modify its activity and the link between *GSK3beta* and HDAC6 involved in neurodegenerative disorders (Chen et al., 2010).

Bilirubin is a reddish yellow pigment generated when the normal red blood cells break. Normal levels range from 0.2 to 1.2 mg/dL (Davis, 2020). In adults, indirect hyperbilirubinemia can be due to overproduction, impaired liver uptake or abnormalities of conjugation (Gondal and Aronsohn, 2016). For AML patients,[[Inline Image]][[Inline Image]] enasidenib is an inhibitor of mutant IDH2 proteins used to treat newly diagnosed mutant-IDH2 AML patients (Pollyea et al., 2019). The most common treatment-related adverse events are indirect hyperbilirubinemia (31%), nausea (23%), and fatigue (Steinwascher et al., 2015). Therefore, bilirubin is an important biomarker for monitoring adverse effect in AML patients who receive treatment.

Preclinical studies have discovered that Smac mimetics can directly cause cancer cell death, or make tumor cells become more sensitive to various cytotoxic treatment agents, including conventional chemotherapy, radiotherapy, or new drugs (Fulda, 2015). There is synergistic interaction of Smac mimetic and HDAC inhibitors in AML cell lines, and Smac mimetic and HDAC inhibitors can trigger necroptosis when caspase activation is blocked (Meng et al., 2016).

AKT.p308 and Src.p527 are phosphorylated signal transduction proteins. These two proteins are found to have lower expression in M0, M1, M2, but they have higher levels in the other AML French-American-British (FAB) types. The expression of those two proteins, together with 22 other proteins, can be used to define distinct signatures for each FAB type (Kornblau et al., 2009).

*PTEN* is a tumor suppressor protein. Promising anti-cancer agents, HDAC inhibitors, particularly trichostatin A (TSA), can promote PTEN membrane translocation. Meng et al. (2016) reveals that non-selective HDAC inhibitors, such as TSA or suberoylanilide hydroxamic acid (SAHA), induces *PTEN* membrane translocation through *PTEN* acetylation at K163 by inhibiting HDAC67. Similarly, treatment with an HDAC6 inhibitor alone promoted *PTEN* membrane translocation and correspondingly dephosphorylated AKT. The combination of celecoxib and an HDAC6 inhibitor synergistically increases *PTEN* membrane translocation and inactivated AKT (Zhang and Gan, 2017).

Our results show that multiple treatments improve efficiency of drugs for curing AML. This can be biologically explained. HDAC inhibitors have emerged as a potent and promising strategy for the treatment of leukemia via inducing differentiation and apoptosis in tumor cells (Jin et al., 2016). A phase II study with 37 refractory acute myelogenous leukemia (AML) patients shows only minimal activity of Vorinostat (HDACi), and Vorinostat fails to control the leukocyte count among most AML patients (Schaefer et al., 2009). A preclinical study reveals that the combination regimen of chidamide (a novel orally active HDAC inhibitor) and IDA could rapidly diminish the tumor burden in patients with refractory or relapsed AML (Li et al., 2017). A Phase II trial of Vorinostat with idarubicin (IDA) and Ara-C for patients with newly diagnosed AML or myelodysplastic syndrome reveals good activity with overall response rates of 85%. No excess toxicity due to Vorinostat is observed (Garcia-Manero et al., 2012). Taken together, HDACs in combination therapy with IDA or other chemotherapeutic drugs show encouraging clinical activity in different hematologic malignancies. This explains that the combination of HDAC and IDA is the best treatment.

Although MGANITE shows remarkable features in ITE for estimation and optimal treatment selection; the results in this paper are very preliminary. Training stable GANs is a challenging task. The training process is inherently unstable, resulting in the inaccurate estimation of ITEs. In this study, we ignore unobserved confounders, unmeasured variables that affect both patients' medical prescription and their outcome. Overlooking the presence of unobserved confounders may lead to biased results. The main purpose of this paper is to stimulate discussion about how to use AI as a powerful tool to improve the estimation of ITEs and optimal treatment selection. We hope that our results will greatly increase the confidence in using AI as a driving force to facilitate the development of precision oncology.

## Data Availability Statement

The data analyzed in this study is subject to the following licenses/restrictions: data were obtained from Department of Biostatistics, The University of Texas MD Anderson Cancer Center. Requests to access these datasets should be directed to Data access need to request from Xuelin Huang, xlhuang@mdanderson.org.

## Ethics Statement

Ethical review and approval was not required for the study on human participants in accordance with the local legislation and institutional requirements. The patients/participants provided their written informed consent to participate in this study.

## Author Contributions

MX and WL: conception and design. QG, MX, and SF: development of methodology. XH: acquisition of data. QG, SG, YL, and SF: analysis and interpretation of data. MX, QG, SG, SF, YL, WL, and XH: writing, review, and/or revision of the manuscript. All authors: contributed to the article and approved the submitted version.

## Conflict of Interest

The authors declare that the research was conducted in the absence of any commercial or financial relationships that could be construed as a potential conflict of interest. The reviewer SJ declared a shared affiliation, with no collaboration, with the authors XH and SF to the handling editor at the time of review.
