# Supplementary material for: Conditional Generative Adversarial Networks for Individualized Treatment Effect Estimation and Treatment Selection
Source: Front Genet. 2020 Dec 11;11:585804. doi: 10.3389/fgene.2020.585804 (PMC7759680; doi:10.3389/fgene.2020.585804)
Supplement: Supplementary file 1 [file Data_Sheet_1.PDF]

Table S1. Top 30 biomarkers identified by Garson algorithm.

Biomarkers	Ri	Sum_Ri	Biomarkers	Ri	Sum_Ri	Biomarkei	Ri	Sum_Ri
CD20	0.0192	0.0192	ALBUMIN	0.0126	0.3766	AKT	0.0110	0.7250
EFS	0.0187	0.0380	INFECTION	0.0124	0.3890	GSK3.p	0.0110	0.7360
CD7	0.0168	0.0547	PB_Blast	0.0122	0.4258	MTOR	0.0109	0.7468
SSBP3	0.0163	0.0711	S6RP.p240.24	0.0121	0.4379	BAD.p136	0.0108	0.7577
BAD.p112	0.0161	0.0871	SURVIVIN	0.0121	0.4500	TP38.p	0.0108	0.7684
CREATININ	0.0159	0.1031	HGB	0.0120	0.4620	TP53	0.0107	0.7791
CD10	0.0158	0.1189	STAT3.p727	0.0120	0.4740	ERk2.p	0.0106	0.7897
BILIRUBIN	0.0157	0.1346	SEX	0.0120	0.4859	BAD.p155	0.0105	0.8002
CG.group	0.0157	0.1503	Source	0.0119	0.4979	STAT5.p4	0.0102	0.8104
CD13	0.0150	0.1653	BAX	0.0119	0.5097	MCL1	0.0101	0.8205
AHD	0.0146	0.1799	TP	0.0118	0.5215	S6.p235	0.0101	0.8306
FAB	0.0141	0.1940	BCAT	0.0118	0.5333	MEK	0.0101	0.8407
RACE	0.0140	0.2080	CD34	0.0118	0.5450	ERK2	0.0101	0.8508
FIBRINOGEI	0.0136	0.2216	XIAP	0.0117	0.5567	SSBP2	0.0100	0.8608
CD33	0.0132	0.2348	TP27	0.0117	0.5684	PTEN.p	0.0100	0.8707
BAD	0.0131	0.2479	MEK.p	0.0116	0.5800	PTEN	0.0099	0.8807
S6	0.0131	0.2609	STAT3	0.0115	0.5915	SRC	0.0099	0.8906
PKCA.p	0.0130	0.2740	ZUBROD.S	0.0113	0.6028	MYC	0.0099	0.9004
Age_at_Dx	0.0129	0.2869	DJI	0.0113	0.6140	SMAC	0.0096	0.9101
NRP1	0.0129	0.2998	PKCA	0.0112	0.6252	ACTB	0.0095	0.9195
PLT	0.0129	0.3127	CCND1	0.0112	0.6364	AKT.p308	0.0094	0.9290
PRIOR_MAI	0.0129	0.3256	GSK3	0.0112	0.6476	BCL2	0.0093	0.9382
WBC	0.0129	0.3385	STAT6.p	0.0111	0.6587	D835	0.0092	0.9475
CD19	0.0128	0.3513	STAT3.p705	0.0111	0.6698	STAT1.p	0.0091	0.9566
PRIOR_CHE	0.0127	0.3640	BM_Blast	0.0111	0.6809	ITD	0.0090	0.9656
LDH	0.0123	0.4013	BAK	0.0111	0.6920	P70S6K.p	0.0089	0.9745
AKT.p473	0.0123	0.4136	PRIOR_XRT	0.0110	0.7030	P70S6K	0.0087	0.9832
PB_Blast	0.0122	0.4258	BCLXL	0.0110	0.7140	SRC.p527	0.0086	0.9919
						MTOR.p	0.0081	1.0000
