# Supplementary material for: Conditional Generative Adversarial Networks for Individualized Treatment Effect Estimation and Treatment Selection
Source: Front Genet. 2020 Dec 11;11:585804. doi: 10.3389/fgene.2020.585804 (PMC7759680; doi:10.3389/fgene.2020.585804)
Supplement: Supplementary file 2 [file Data_Sheet_2.docx]

**Supplementary Note A**

**Algorithms**

To solve the optimization problems (4) and (7), we first need to use sampling formulas to approximate the expectations. We first discuss implementation of the imputation block.

*Imputation block optimization*

Assume that $n$ individuals are sampled. Sampling approximation of $V(D_{G}, G)$ is given by

$\hat{V}(D_{G}, G)\approx\frac{1}{n}\sum_{i=1}^{n} {[M}_{i}^{T}\log\left( D_{G}(X^{\left( i \right)}, T^{\left( i \right)}, \tilde{Y}^{\left( i \right)}, M_{i},\theta_{d} \right)+(\boldsymbol{1}-M_{i})^{T}\log(\boldsymbol{1}-D_{G}\left( X^{\left( i \right)}, T^{\left( i \right)}, \tilde{Y}^{\left( i \right)}, M_{i},\theta_{d} \right))]$, (A1)

where $\tilde{Y}=G\left( X,Y_{f},{T\odot M, (\boldsymbol{1}-M)\odot Z}_{G},\theta_{G} \right).$

To enforce that the estimated factual outcome $\tilde{Y}_{\eta}^{(i)}$should be as close to the observed factual outcome $Y_{f}^{(i)}$ as possible (2), we post the following restriction:

$l(G)=\frac{1}{n}\sum_{i=1}^{n} (Y_{f}^{\left( i \right)}-\tilde{Y}_{\eta}^{(i)})^{2}$. (A2)

The optimization problem (4) can be implemented by

$\min_{D_{G}} -\hat{V}(D_{G}, G)$, (A3)

$\min_{G} \hat{V}\left( D_{G}, G \right)+\lambda l(G)$. (A4)

Optimization problems (A3) and (A4) can be solved by backpropagation (stochastic gradient decent) algorithms (4). The details for the algorithms are given in supplementary note B.

**ITE block optimization**

ITE block intends to estimate the counterfactual outcomes using the observed outcomes and imputed counterfactual outcomes. Its performance metrics are defined

for $K=2$ (binary treatments):

$L\left( G_{I} \right)=\frac{1}{n}\sum_{i=1}^{n} [\left( \bar{y}_{1}^{\left( i \right)}-\bar{y}_{0}^{\left( i \right)} \right)-(\hat{y}_{1}^{\left( i \right)}-\hat{y}_{0}^{\left( i \right)}))^{2}]$ , (A5)

for $K>2$:

$L\left( G_{I} \right)=\frac{1}{n}\sum_{i=1}^{n} ||{\bar{\boldsymbol{y}}}^{\left( i \right)}-{\hat{\boldsymbol{y}}}^{(i)}{||}_{2}^{2}$. (A6)

Sampling formula for $V_{I}\left( D_{I}, G_{I} \right)$ is

$\hat{V}_{I}\left( D_{I}, G_{I} \right)=\frac{1}{n}\sum_{i=1}^{n} [\log D_{I}(x^{\left( i \right)}, \left( y^{*})^{\left( i \right)} \right)+\log(1-D_{I}\left( x^{\left( i \right)}, \left( y^{*})^{\left( i \right)} \right) \right)]$. (A7)

The optimization problem (7) for ITE can be reformulated as

$\min_{D_{I}} -\hat{V}_{I}\left( D_{I}, G_{I} \right)$, (A8)

$\min_{G_{I}} \hat{V}_{I}\left( D_{I}, G_{I} \right)+\gamma L\left( G_{I} \right)$. (A9).

Again, stochastic gradient descent methods can be used to solve optimization problems (A8) and (A9). Algorithms for their numerical implementation are similar to algorithms for the imputation block.
