# Supplementary material for: Conditional Generative Adversarial Networks for Individualized Treatment Effect Estimation and Treatment Selection
Source: Front Genet. 2020 Dec 11;11:585804. doi: 10.3389/fgene.2020.585804 (PMC7759680; doi:10.3389/fgene.2020.585804)
Supplement: Supplementary file 3 [file Data_Sheet_3.docx]

**Supplementary Note B**

**Algorithms for implementing stochastic gradient decent**

Algorithm is a modified version of Algorithms in (4).

Let $l$ be the number of steps to apply to the discriminator.

**for** number of training iterations **do**

**for** $l$ steps **do**

1. Sampling minibatch of $m$ noise vectors $\left\{ z^{(1)},\ldots,z^{(m)} \right\}$ from prior $P_{g}(z)$.
2. Sampling minibatch of $m$ samples of feature, observed outcomes, treatment and treatment assignment indicator data $\left\{ X^{(i)}, T^{(i)}, M^{(i)}, y_{f}^{(i)}, i=1,\ldots,m \right\}$from data generating distributions.
3. Update the parameters in discriminator by descending its stochastic gradient:

$-\nabla_{\theta_{d}}\hat{V}(D_{G},G,\theta_{d})$ . (B1)

**end if**

1. Sampling minibatch of $m$ noise vectors $\left\{ z^{(1)},\ldots,z^{(m)} \right\}$ from prior $P_{g}(z)$.
2. Update the parameters in generator by descending its stochastic gradient:

$\nabla_{\theta_{g}}\hat{V}\left( D_{G},G\left( \theta_{g} \right) \right)+\lambda l(G\left( \theta_{g} \right))$. (B2)

**end if**
